# Fast and Accurate ROI Extraction for Non-Contact Dorsal Hand Vein Detection in Complex Backgrounds Based on Improved U-Net

**DOI:** 10.3390/s23104625

**Published:** 2023-05-10

**Authors:** Rongwen Zhang, Xiangqun Zou, Xiaoling Deng, Ziyang Wang, Yifan Chen, Chengrui Lin, Hongxin Xing, Fen Dai

**Affiliations:** 1 College of Electronic Engineering (College of Artifificial Intelligence), South China Agricultural University, Guangzhou 510642, China; 201721110327@stu.scau.edu.cn (R.Z.); dengxl@scau.edu.cn (X.D.); 20213162184wzy@stu.scau.edu.cn (Z.W.); 1049764125@stu.scau.edu.cn (Y.C.); linchengrui@stu.scau.edu.cn (C.L.); x_hongxin@stu.scau.edu.cn (H.X.); 2 Guangzhou Intelligence Oriented Technology Co., Ltd., No. 604, Tian’an Technology Development Building, Tian’an Hi-Tech Ecological Park, No. 555, North Panyu Avenue, Panyu District, Guangzhou 511493, China; nkwavelet@163.com; 3 Lingnan Modern Agriculture Guangdong Laboratory, Guangzhou 510642, China; 4 National International Joint Research Center of Precision Agriculture Aviation Application Technology, Guangzhou 510642, China

**Keywords:** deep learning, contactless dorsal hand vein, keypoint detection, region of interest (ROI), lightweight

## Abstract

In response to the difficulty of traditional image processing methods to quickly and accurately extract regions of interest from non-contact dorsal hand vein images in complex backgrounds, this study proposes a model based on an improved U-Net for dorsal hand keypoint detection. The residual module was added to the downsampling path of the U-Net network to solve the model degradation problem and improve the feature information extraction ability of the network; the Jensen–Shannon (JS) divergence loss function was used to supervise the final feature map distribution so that the output feature map tended to Gaussian distribution and improved the feature map multi-peak problem; and Soft-argmax is used to calculate the keypoint coordinates of the final feature map to realize end-to-end training. The experimental results showed that the accuracy of the improved U-Net network model reached 98.6%, which was 1% better than the original U-Net network model; the improved U-Net network model file was only 1.16 M, which achieved a higher accuracy than the original U-Net network model with significantly reduced model parameters. Therefore, the improved U-Net model in this study can realize dorsal hand keypoint detection (for region of interest extraction) for non-contact dorsal hand vein images and is suitable for practical deployment in low-resource platforms such as edge-embedded systems.

## 1. Introduction

With the rapid development of security systems and the internet economy, personal identification has become an essential technology for security purposes. Furthermore, due to the outbreak of seasonal influenza, face recognition requires the removal of masks and fingerprints and palm prints require contact with the equipment, all of which increase the chances of infection with the virus. Venous structures are vascular structures that exist under the skin and are difficult to damage or replicate. The advantage of vein technology is that it can only be obtained from a living person and the pattern does not change over time. As a result, vein patterns provide more accurate results as well as a higher level of safety. The proposed dorsal hand vein (DHV) recognition system is therefore contactless, low cost, and more secure than other popular biometric systems.

In recent years, DHV recognition has gained much attention as an emerging biometric technology. Owing to its safety, accuracy, and effectiveness, more and more researchers are involved [1,2,3,4]. Lefkovits et al. [5] presented a dorsal hand vein recognition method based on convolutional neural networks (CNN). Wang et al. [6] put forward the method of dorsal hand vein recognition based on bit plane and block mutual information. Chin et al. [7] described dorsal hand vein recognition using statistical and Gray Level Co-occurrence Matrix (GLCM)-based features extraction techniques and artificial neural networks (ANNs). Liu et al. [8] presented an improved biometric graph matching method that included edge attributes for graph registration and a matching module to extract discriminating features. Sayed et al. [9] proposed a dorsal hand recognition system working in real time to achieve good results with a high frame rate.

It is well known that DHV recognition belongs to the family of hand-based biometrics. DHV recognition is a technology that uses analysis and matching of the subcutaneous vein structure on the dorsal hand to achieve personnel identity verification. Region of interest (ROI) extraction is a key step in DHV recognition. Lin et al. [10,11,12] binarized an image of an open hand, calculated the Euclidean distance between each edge pixel of the hand and the midpoint of the wrist, and used these distances to construct a distance distribution map with a shape very similar to the geometry of the dorsal hand; they selected the second and fourth finger webs as reference points to define a square ROI. Damak et al. [13] used the Otsu thresholding method for hand segmentation, hand boundary tracing, drawing hand boundary distance contours by scanning contours, and rotating the image so that the line connecting the first and third finger valleys became horizontal by selecting four hand boundaries (vertical left limit, vertical right limit, horizontal lower limit, and horizontal upper limit) to create the ROI region. Cimen [14] split the hand image and determined the boundaries of the hand surface area. The entire image was then scanned pixel by pixel from right to left and top to bottom; the first point that reached 255 pixels was found to be the tip of the bone, at which point a square area of 256 × 256 pixels was selected 150 pixels down as the ROI. Meng et al. [15,16,17,18,19,20] converted the image of a clenched hand into a binary image and used morphological methods to detect the dorsal boundary of the hand; after determining the distance between each point on the boundary and the midpoint of the wrist, the valley points between the fingers were found to be the corresponding valley points in the distance contour. A final fixed-size sub-image based on valley points 1 and 3 was extracted. A total of 240 images of 80 users were obtained from the Bosphorus Hand Vein Database [21]. Nozaripour et al. [22] used the sparse representation, kernel trick, and a different technique of the region of interest (ROI) extraction that was present in the previous work, and a new and robust method against rotation was introduced for dorsal hand vein recognition. A general ROI extraction algorithm usually consists of the following main steps: (1) converting the hand image to a binary image with a segmentation algorithm; (2) performing hand boundary tracking; (3) calculating the distance contour points (points located on the hand contour) and reference points between (the midpoint of the wrist is generally used as the reference point); (4) locating the ROI based on the detected points; and (5) cropping the ROI sub-image.

The current research objects were dorsal hand vein images acquired under constrained environments with restricted hand positions and very clean backgrounds. Traditional image processing algorithms are very favorable for segmenting hands in such images; however, these algorithms struggle to segment complete hand images in complex backgrounds, which limits their practical applications. So far, deep learning techniques have not been studied for ROI extraction of vein images of the dorsal hand. Today, deep learning has become one of the most important techniques in the field of computer vision. To date, many classical neural networks [23,24,25,26,27,28] have been proposed, and impressive results have been achieved in many recognition tasks.

Keypoint detection research is divided into two methods: one is to directly regress the keypoint coordinates through the fully connected layer of the neural network, and the other is to directly output the heat map by removing the fully connected layer of the neural network; the coordinates corresponding to the peak of the heat map are the keypoint coordinates. Heat map-based keypoint regression is a computer vision technique that involves generating a heat map of an image to highlight the presence or absence of specific features and predict their locations. In this approach, each feature (or keypoint) is represented as a Gaussian function centered at its true location, and a heat map is produced by summing together these Gaussian functions. The resulting heat map can be used to train a convolutional neural network model to perform keypoint detection and regression tasks on new images. Heat map regression can also be combined with color encoding to visually highlight targets and relevant keypoints in the image based on their confidence scores [29]. Most face keypoint detection uses the method of direct regression of keypoint coordinates from the fully connected layer [30,31] because the face can be regarded as a rigid body, the relative position between points is constant, the spatial information of the feature map is lost, and the spatial generalization is lacking. While the hand is very flexible and has a more diverse posture when collecting non-contact dorsal hand vein images because there is no hand fixation device, the hand will be pitched, bent, and opened and closed differently, and the relative positions of keypoints will vary greatly, so this study used the heat map method to detect the keypoints of dorsal hand vein images. A U-Net [32] network model was used as the classical model in the fully convolutional neural network and divided into downsampling and upsampling paths, and the low-level features were fused with high-level features through jumping connections to obtain richer feature information, which could detect the keypoints of dorsal hand vein images more accurately.

Deep learning is developing rapidly in the field of computer vision and has better capabilities than traditional image processing algorithms for image detection and recognition in complex situations, so it is crucial to systematically study deep learning for ROI extraction in keypoint detection of non-contact dorsal hand vein images. To this end, this study constructed a dorsal hand vein dataset by capturing unconstrained dorsal hand vein images with a self-developed infrared image acquisition device and proposed an improved U-Net network model, which added residual modules [33] to the downsampling path of the original U-Net network to solve the problem of model degradation caused by deepening the network. We also replaced the transpose convolution [34] in the upsampling path of the U-Net network with bilinear interpolation [35] to avoid “checkerboard artifacts”. To ensure the final output feature map to tend toward Gaussian distribution, we introduced the Jensen–Shannon (JS) divergence loss function as supervision. Finally, Soft-argmax [36] was used to decode the keypoints’ coordinates from the feature map for end-to-end training. We utilized the improved network for hand dorsum vein image keypoint detection and then extracted the ROI based on the keypoints.

## 2. Materials and Methods

### 2.1. Experimental System

**Figure 1:**
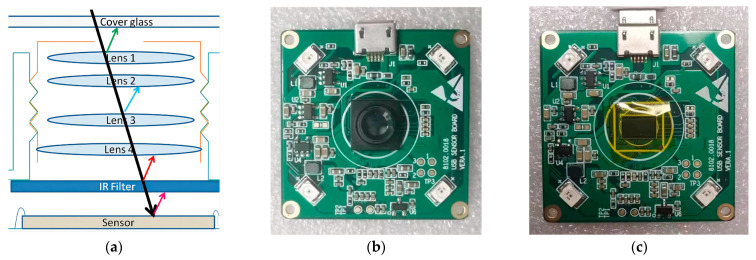
Camera hardware structure: (**a**) basic structure of a camera; (**b**) camera hardware; (**c**) CMOS sensor under the lens.

When near-infrared light penetrates through the skin tissue, it is absorbed by the hemoglobin in the blood due to its high absorption rate for near-infrared light. As a result, when near-infrared light is irradiated onto the hand vein, the blood in the vein partially absorbs it, and the remaining unabsorbed light is scattered back to the imaging device. By using the scattered light signal received by the imaging device, the characteristic image of the hand vein can be reconstructed through processing algorithms. Figure 1a shows the basic structure of a camera with an infrared filter between the lens and a Complementary Metal–Oxide–Semiconductor (CMOS) sensor. Figure 1b depicts an unpackaged camera with four infrared LEDs, and Figure 1c shows the CMOS sensor without the lens attached. A CMOS sensor is a type of image sensor commonly used in digital cameras and other imaging devices. The basic principle behind the CMOS sensor’s imaging capability is that it is made up of millions of tiny pixels, each of which collects and converts light into an electrical charge. When light enters the camera lens, it is focused onto the surface of the CMOS sensor. Each pixel in the sensor contains a photodiode that converts photons of light into electrons. These electrons are then stored in a capacitor that is attached to each photodiode. The amount of charge stored in each capacitor is proportional to the amount of light that fell on the corresponding pixel. The CMOS sensor then reads the charge from each capacitor and converts it into a digital value that represents the brightness of the pixel. Once all the pixels have been read, they are combined to create a single digital image.

**Figure 2:**
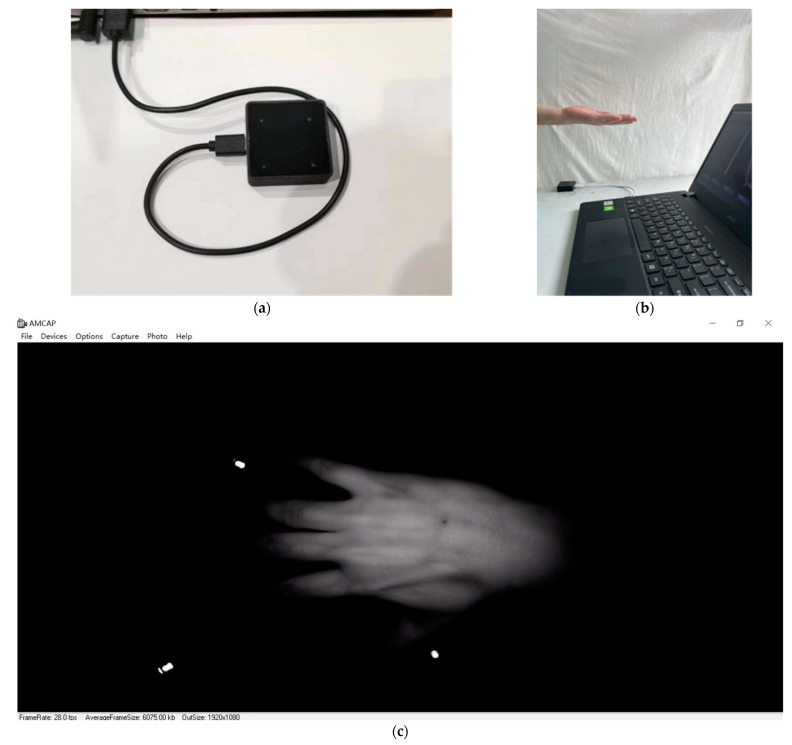
Self-built dorsal hand vein image dataset acquisition process: (**a**) acquisition device; (**b**) acquisition site; (**c**) acquisition software interface.

The infrared camera was placed flat on a desk with the infrared camera pointed upwards and connected to the computer via a data cable, the hand was positioned 10~15 cm from the infrared camera with no restriction on the rotation angle, and the image of the vein on the dorsal hand was captured using the open source software Amcap. The acquisition equipment, acquisition site plan, and acquisition software interface are shown in Figure 2.

### 2.2. Datasets

#### Self-Built Dataset and Jilin University Public Dataset

**Figure 3:**
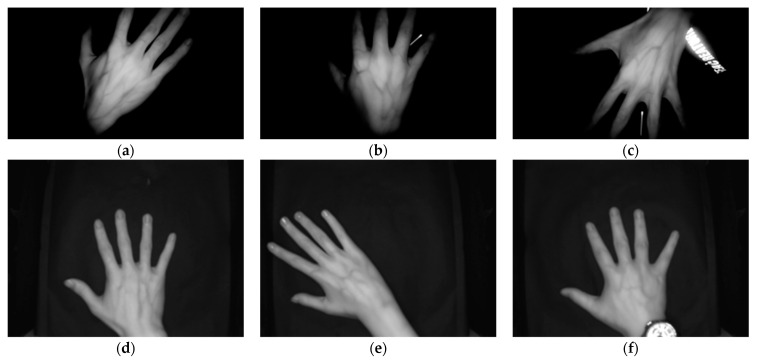
Selected sample images from the self-constructed dorsal hand vein image dataset and Jilin University public dataset. Self-built dataset: (**a**) clean background; (**b**) background with fluorescent lamp; (**c**) background with the subject’s clothing pattern. Jilin University public dataset: (**d**) placed in the center without any jewelry; (**e**) placed off-center; (**f**) wearing a wristwatch.

In this study, NIR imaging was used to acquire images of the veins on the dorsal hand. A NIR light source was used to illuminate the surface of the subject’s right and left hands, and an 850 nm filter was mounted in front of the camera lens to prevent visible light from entering the camera. The current supply to the acquisition system was kept constant throughout the experiment. A self-built dataset of dorsal hand vein images was acquired at South China Agricultural University, and the researchers placed no restrictions on the angle, distance, or background of the subject’s hand. A total of 5 images were taken of each individual’s open left and right hand using an image size of 1920 pixels × 1080 pixels, resulting in a dataset of 1890 unconstrained dorsal hand vein images from 189 individuals with a clean background, background interference, incomplete occlusion, and wearing rings, etc. Some of the sample images are shown in Figure 3a–c. The Jilin University public dataset [8] acquisition infrared device was facing downwards, and the hand was flat on a clean black platform. The dataset collected 222 people, and each right hand was imaged 10 times using an image size of 640 pixels × 480 pixels for a total of 2220 dorsal hand vein images. Some sample images are shown in Figure 3d–f.

### 2.3. Improved U-Net Model for Keypoint Detection in Dorsal Hand Vein Images

**Figure 4:**
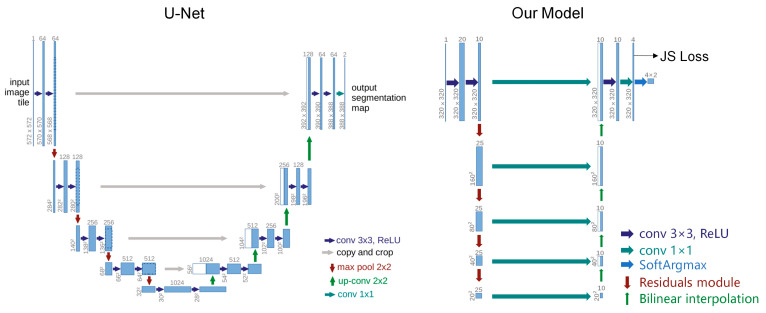
U-Net and improved U-Net network structure.

The improved U-Net network structure is shown in Figure 4.

#### 2.3.1. Residual Module for Downsampling

**Figure 5:**
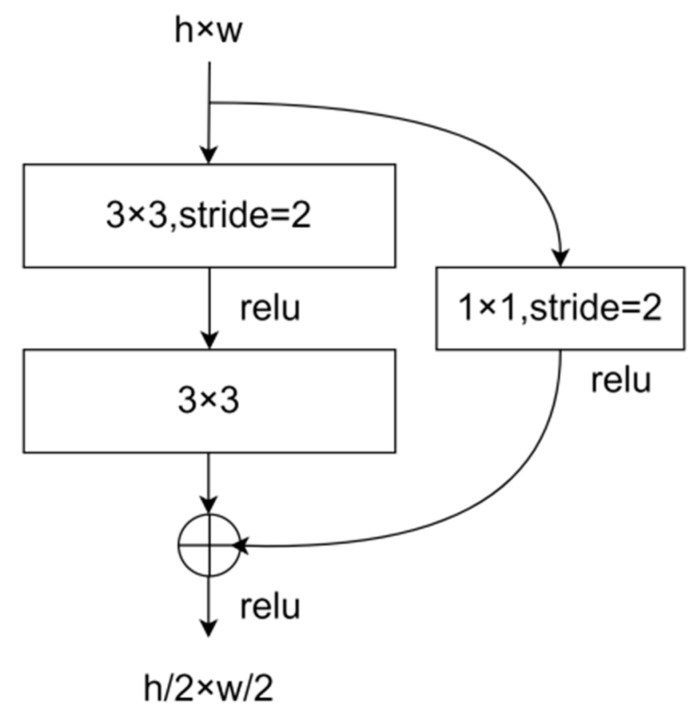
Residual module used in the improved U-Net network.

The existing U-Net network model used ordinary convolutional layers for downsampling, and network degradation occurred when the network was deepened. Using the residual module could avoid gradient disappearance and explosion to solve the network degradation problem. For the first layer of the downsampling path, a convolutional kernel of size 3 × 3 was used to expand the 1-channel image to 20 channels, a convolutional kernel of size 3 × 3 was used to reduce the 20-channel image to 10 channels, and then edge fill was used to ensure that the feature maps were of the same size. The first layer of the residual module was a convolutional (Figure 5) kernel with a size of 3 × 3 and a stride size of 2 pixels to achieve downsampling to reduce the feature map length and width by 1/2. The short-circuit connection used a convolutional kernel with a size of 1 × 1 and a stride size of 2 pixels to adjust the number of channels and size of the feature map to ensure that the outputs of the two paths could be added together with the same number of channels and size. The downsampling paths were downsampled using a total of four residual modules, and the feature map size was reduced to 1/16 of the original input image length and width.

#### 2.3.2. Bilinear Interpolation for Upsampling

The existing U-Net network model used transpose convolution for upsampling, which increased the feature map size by inserting zeros, and then performed convolution to achieve upsampling. However, due to the lack of sub-pixel interpolation, it was prone to cause “checkerboard artifacts”. Bilinear interpolation [35] is a common image interpolation algorithm used to upscale low-resolution images to high-resolution ones. It is based on four nearest neighbor pixels to interpolate using a weighted average method to calculate the grayscale value of the target pixel, therefore, the upsampling path used bilinear interpolation to replace transposed convolution for up-sampling. The downsampled output feature map of the corresponding layer was first convolved using a convolution kernel with a size of 1 × 1 so that the number of channels of the downsampled output feature map was the same as that of the corresponding upsampled layer output feature map, thus directly adding up to achieve the fusion of low-level and high-level features to further enrich the detailed features of the feature map.

#### 2.3.3. Jensen–Shannon (JS) Divergence Loss

The ground truth heat map followed a Gaussian distribution, and only the Mean Squared Error (MSE) loss function was used for supervised training. The output heat map only converged to the labeled heat map in terms of value and not in terms of shape. The network model outputted a 4-channel heat map, each channel heat map corresponded to a keypoint, the heat map could be regarded as a probability distribution, and there were multiple peaks on the heat map during training. The Kullback–Leibler (*KL*) divergence is a measure of how similar two probability distributions are; the more similar the two probability distributions are, the smaller the *KL* divergence is, and it is zero if they are exactly equal. The *JS* divergence is a variant of the *KL* divergence and solves the *KL* divergence asymmetry problem. Consider Equations (1) and (2) where *P* and *Q* are two probability distributions; *x_i_* is the *i*-th component; and *P(x_i_)* and *Q(x_i_)* denote the probability values associated with the *i*-th component in probability distributions *P* and *Q*, respectively; n is the number of components; *KL(P‖Q)* represents the *KL* divergence between distributions *P* and *Q*; and *JS(P‖Q)* represents the *JS* divergence between distributions *P* and *Q.*

(1) $$\begin{array}{c} KL\left(P∥Q\right)=\sum_{i=1}^{n}P\left(x_{i}\right)\log\frac{P\left(x_{i}\right)}{Q\left(x_{i}\right)} \end{array}$$

(2) $$\begin{array}{c} JL\left(P∥Q\right)=\frac{1}{2}KL\left(P∥\frac{P+Q}{2}\right)+\frac{1}{2}KL\left(Q∥\frac{P+Q}{2}\right) \end{array}$$

#### 2.3.4. Soft-Argmax to Obtain Keypoint Coordinates

Some studies [37,38,39] used the MSE loss function to calculate the loss value between the Gaussian heat map labels and the neural network’s output feature maps, through which supervised learning was applied to achieve keypoint detection using argmax to calculate the index of the maximum response point. The operation of selecting the point with the highest probability on a heatmap as the model’s prediction result is known as argmax. However, since the argmax operation is non-differentiable, gradients cannot be passed to it during model training. As a result, the predicted coordinates can only be obtained offline, making end-to-end training impossible. In contrast, Soft-argmax is differentiable, which allows direct connection to the network’s feature map output layer to compute the index of the maximum response point using the L2 loss function to calculate the loss value between the key point coordinate labels and the index of the maximum response point for supervised learning, enabling end-to-end training. Equation (3) represents the softmax function, while Equation (4) represents the Soft-argmax function [36].

(3) $$\begin{array}{c} softmax\left(x\right)=\frac{e^{x_{i}}}{\sum_{j}e^{x_{j}}} \end{array}$$

(4) $$\begin{array}{c} Softargmax\left(x\right)=\sum_{i}\frac{e^{{\beta x}_{i}}}{\sum_{j}e^{{\beta x}_{j}}}i \end{array}$$

### 2.4. ROI Calculation Method

**Figure 6:**
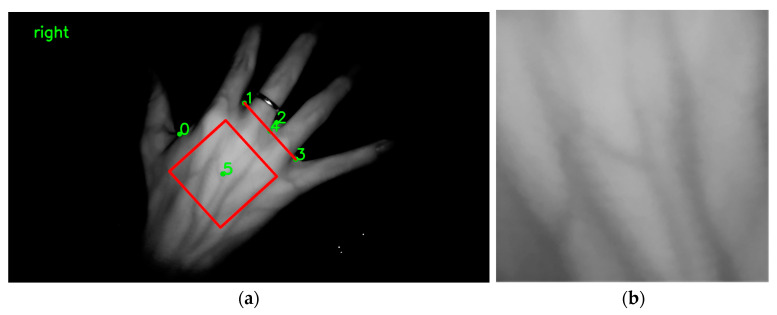
Self-built dorsal hand vein image dataset region of interest extraction method: (**a**) keypoint detection and region of interest selection for the dorsal hand vein image; (**b**) region of interest image adjusted using linear interpolation.

The Pipeline of DHV ROI Extraction is summarized in Algorithm 1 as shown in Figure 6.
**Algorithm 1** Overall Pipeline of DHV ROI Extraction**Require:** A DHV image **I** and our detector ***D***.1: Feed **I** to ***D*** to obtain four keypoints P_0_, P_1_, P_2_, P_3_;2: Set the line through P_1_P_3_ as the X-axis;3: Set the direction perpendicular to P_1_P_3_ and P_0_ as the positive direction of the Y-axis;4: Set the midpoint P_4_ of P_1_P_3_ as the origin (x_0_, y_0_);5: Take $\left\|P_{1}P_{3}\right\|$ as the unit length of axis;6: Construct the local coordinate system;7: Set the center coordinate P_5_ of the *ROI* to $\left(x_{0},y_{0}+\frac{5}{6}\left\|P_{1}P_{3}\right\|\right)$;8: Set the side length of *ROI* to $\left\|P_{1}P_{3}\right\|$;9: **return** segmented *ROI*

### 2.5. Model Evaluation Indicators

The equations for calculating model evaluation metrics are as follows:

(5) $$\begin{array}{c} d=\sqrt{{\left(x_{1}-x_{2}\right)}^{2}+{\left(y_{1}-y_{2}\right)}^{2}} \end{array}$$

(6) $$\begin{array}{c} f\left(x_{i}\right)=\left\{\begin{array}{c} 1,d<40\\ 0,d\geq 40 \end{array}\right. \end{array}$$

(7) $$\begin{array}{c} accuracy=\frac{\sum_{i=0}^{n}f\left(x_{i}\right)}{n} \end{array}$$

In Equation (5), *d* is the Euclidean distance between the predicted coordinates (*x*_1_*, y*_1_) and the label coordinates (*x*_2_, *y*_2_). In Equation (6), *x_i_* is the *i*-th keypoint. If *d* is less than 40 pixels, it is considered a correct prediction and *f(x_i_)* is set to 1. Otherwise, *f(x_i_)* is set to 0. Equation (7) calculates the accuracy, where n is the total number of predicted keypoints. The accuracy is the proportion of correctly predicted keypoints to all predicted keypoints.

## 3. Experimental Results and Analysis

### 3.1. Model Training

**Figure 7:**
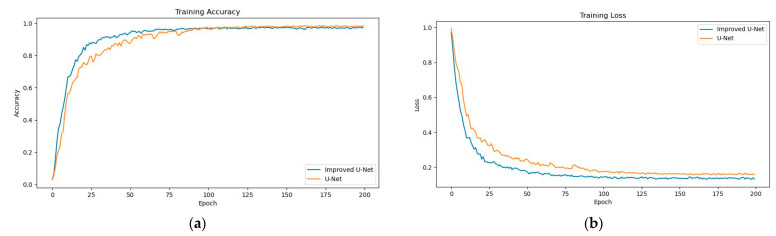
Training accuracy and loss curves for U-Net and improved U-Net: (**a**) training accuracy change curve; (**b**) training loss value change curve.

The training server environment for this study was the Windows 10 64-bit operating system, an Intel(R) Core I7-10700F CPU with 32 GB RAM, and an NVIDIA Quadro RTX5000 graphics card using Python and PyTorch frameworks. From the collected 2200 images, 1100 images were randomly selected and the 4 keypoints of the images were annotated using the open source data annotation software Labelme, randomly divided into training and test sets at a ratio of 8:2, and trained using a batch training size of 32. We selected the Adam optimization method with an initial learning rate of 0.001 and used the equal interval adjustment learning rate strategy (StepLR); the total number of training cycles epoch total = 200. During the training process, data augmentation methods such as horizontal and vertical flipping of images as well as random adjustments to brightness, contrast, and histogram equalization were applied to improve the model’s generalization ability. The training loss function was the average of the MSE loss, JS divergence loss function, and Euclidean distance loss function. The average accuracy and the loss value change curve of the model during training are shown in Figure 7.

### 3.2. Residual Module to Implement Downsampling Comparison Tests

To evaluate whether the residual module resulted in an improvement in the performance of the network model, a modified U-Net model with and without short-circuit connections was used for training.

(1) Qualitative comparison

**Figure 8:**
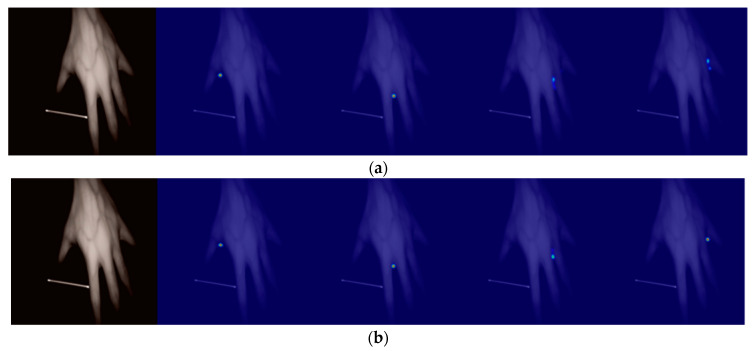
Heat diagram obtained by improving the U-Net model with and without short-circuit connections: (**a**) heat map without short-circuit connections; (**b**) heat map with short-circuit connections.

Using a network model without short-circuit connections for training, the heat map obtained when the dorsal hand was at a certain pitch angle was not very accurate, and there were two points where the heat map did not tend to be Gaussian, so a residual module with short-circuit connections was used to obtain a more accurate heat map as shown in Figure 8.

(2) Quantitative comparison

Tests were carried out on the self-built dataset; the results are shown in Table 1.

In Table 1, we can see that when using the network model without short-circuit connections for training, the model accuracy was 0.6 percentage points lower than that with short-circuit connections, the inference time was reduced by about 5 ms, and the model was reduced by 0.02 M. This was because the short-circuit connection path had a convolutional layer that consumed some inference time, while the short-circuit connection could extract richer feature information, solve the gradient disappearance problem, and improve the model accuracy.

### 3.3. Improved Transposed Convolution as a Bilinear Interpolation Comparison Test

To evaluate whether improving transposed convolution to bilinear interpolation resulted in an improvement in the performance of the network model, a modified U-Net model using transposed convolution and bilinear interpolation was trained.

(1) Qualitative comparison

**Figure 9:**
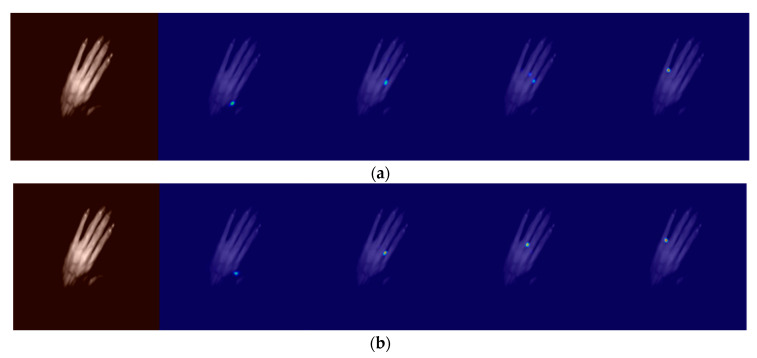
Heat maps obtained by different upsampling methods for the improved U-Net model: (**a**) heat map for transposed convolution; (**b**) heat map for bilinear interpolation.

Using a transposed convolutional network model for training, when the fingers were not open enough, they were misjudged to be the next finger, so bilinear interpolation was used for upsampling and then the output heat map was more accurate, as shown in Figure 9.

(2) Quantitative comparison

Tests were carried out on the self-built dataset; the results are shown in Table 2.

This was because the convolution operation of transposed convolution consumed a certain amount of inference time, and there was no sub-pixel interpolation when the convolution was carried out after interpolating the null and increasing the size of the feature map to achieve up-sampling, whereas bilinear interpolation is a sub-pixel interpolation, and the color transition will be more natural and improve the model accuracy.

### 3.4. Comparative Tests of Supervision Effects with Different Losses

To evaluate whether model training using a Gaussian distribution of the JS divergence loss function supervised feature maps resulted in an improvement in the performance of the network model, supervised training was performed on a modified U-Net model using different loss functions.

(1) Qualitative comparison

**Figure 10:**
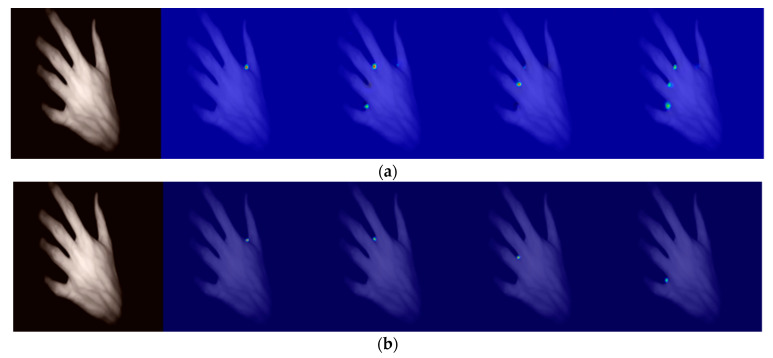
Heat map obtained by improving the U-Net model trained with different losses: (**a**) using only Mean Squared Error (MSE) Loss; (**b**) using multiple losses: MSE loss + Jensen–Shannon divergence loss + Euclidean distance loss.

When only the MSE loss was used for supervised training, the feature map would show multiple peaks, which affected the judgement of peaks, so the MSE loss, JS divergence loss function, and Euclidean distance loss function were used for jointly supervised training to solve the problem of multiple peaks in the heat map, the details of which are shown in Figure 10.

(2) Quantitative comparison

Tests were carried out on the self-built dataset; the results are shown in Table 3.

As shown in Table 3, when only the MSE loss was used for supervised training, the model’s accuracy was 92.3%. When only the JS divergence loss function was used to supervise the probability distribution of the feature maps, the model accuracy was 95%. When only the Euclidean distance loss function was used to supervise the coordinate points, the model accuracy is 95.5%. By simultaneously supervising the feature map array and probability distribution in addition to the coordinate points, the model accuracy was improved by 3.1 percentage points.

### 3.5. Comparison Test of the U-Net Model before and after the Improvement

**Figure 11:**
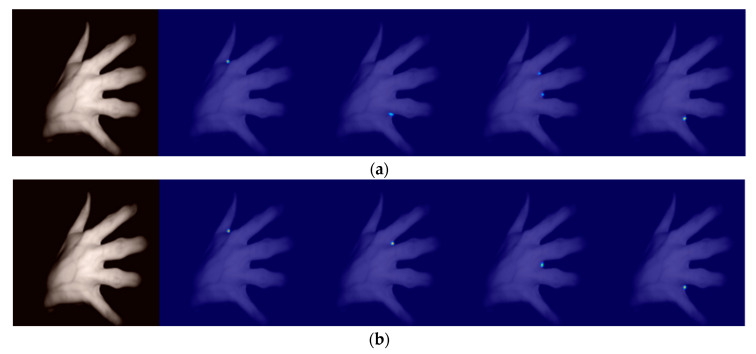
Heat diagrams obtained from the U-Net model before and after the improvement: (**a**) heat map obtained from U-Net; (**b**) heat map obtained from improved U-Net.

To be able to deploy the network model on platforms with limited computing resources (such as embedded systems) for practical applications, the performance of the model before and after the improvement had to be evaluated. The inference time, accuracy, and model size obtained using the improved U-Net network and the original U-Net network trained with the same training set and loss and tested on the same test set are shown in Table 4, and the heat map obtained is shown in Figure 11.

**Figure 12:**
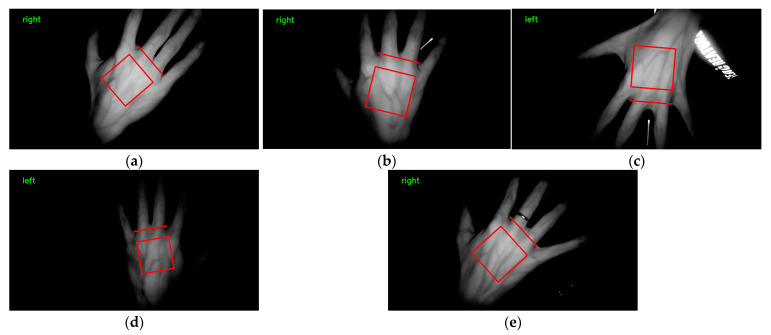
Results of testing the improved U-Net model against the self-built dataset: (**a**) clean background; (**b**) background with fluorescent light; (**c**) background with the subject’s clothing pattern; (**d**) incomplete masking; (**e**) wearing a ring.

Table 4 shows that the accuracy of the original U-Net network model was 97.6% and the model file is 35.0 M, which would occupy more embedded system memory and is not suitable for deployment on embedded systems, while the accuracy of the improved U-Net network model was improved by 1 percentage point and the model file was 1.16 M, which can be deployed on embedded systems for practical applications. The total Floating Point Operations per Second (FLOPs) of U-Net was 7.44 G, while that of the improved U-Net was only 939.0 M. Total FLOPs refers to the total number of floating-point operations performed during a single forward pass of the entire model. A smaller total FLOPs meant that the model required fewer computational resources to perform the same number of computations, resulting in faster execution and requiring fewer computing resources (such as CPU, GPU, etc.) for training or inference. This was because the residual module avoided gradient explosion and disappearance, solved the problem of deep network model degradation, and enabled the network to extract rich feature information despite the significant reduction in model parameters, allowing the model size to be reduced to nearly 1/30 that of the original model and still achieve a higher accuracy than the original U-Net network model. The results of the improved U-Net model for dorsal hand keypoint detection and dorsal hand vein image ROI extraction based on keypoints are shown in Figure 12. It can be seen in the figure that the improved U-Net model could achieve higher accuracy in locating keypoints on the dorsal hand in complex backgrounds.

**Figure 13:**
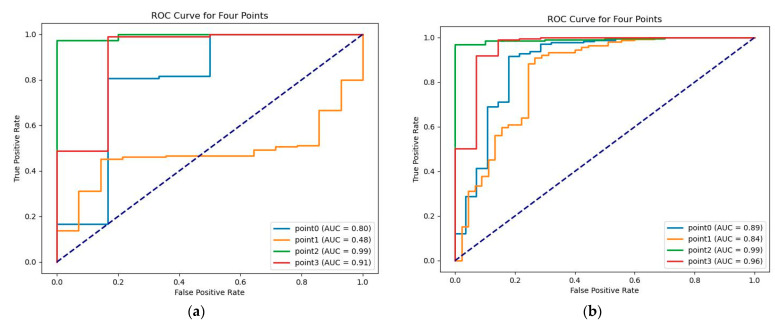
Receiver operating characteristic (ROC) curves for keypoint classification in U-Net and improved U-Net: (**a**) U-Net; (**b**) improved U-Net.

The values on the heatmap corresponded to the confidence that a given point was a keypoint, where higher confidence indicated a higher probability of being a keypoint. Therefore, the predicted values on the heatmap corresponding to the keypoints were outputted, and the model’s ability to classify keypoints before and after improvement was evaluated using receiver operating characteristic (ROC) analysis as shown in Figure 13. It can be seen in Figure 13 that the model’s ability to classify keypoints was stronger after improvement.

### 3.6. Improved U-NET Test Results on the Jilin University Public Dataset

**Figure 14:**
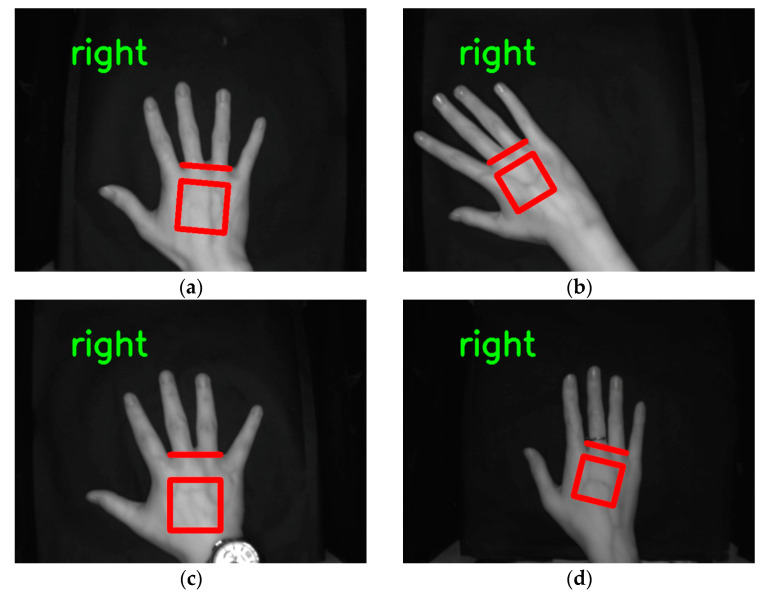
Results of improved U-Net model testing on the Jilin University public dataset: (**a**) no ornaments placed squarely in the middle; (**b**) offset placement; (**c**) wearing a watch; (**d**) wearing a ring.

The network model trained directly on the self-built dataset was tested on 220 images from the Jilin University dataset with an accuracy of 80%, and the test accuracy was 98.1% when trained directly on the Jilin University public dataset. While using a mixture of 1100 self-built dataset images and 300 Jilin University public dataset images as the training set and 220 Jilin University public dataset images as the test set, other training parameters and strategies remained unchanged: the accuracy on the test set was 99.5%, and the test results of ROI extraction of dorsal hand vein images on the Jilin University public dataset are shown in Figure 14. Because of the large differences between the self-built dataset and the Jilin University public dataset (the Jilin University public dataset has a low resolution of 640 × 480 pixels and the dorsal hand is located in the center of the image), the self-built dataset lacked image data similar to the Jilin University public dataset. By supplementing the self-built dataset with a small number of images from the Jilin University public dataset, the trained network model could achieve a high accuracy on both datasets.

### 3.7. Comparison of Conventional Image Processing Methods for Locating Keypoints

**Figure 15:**
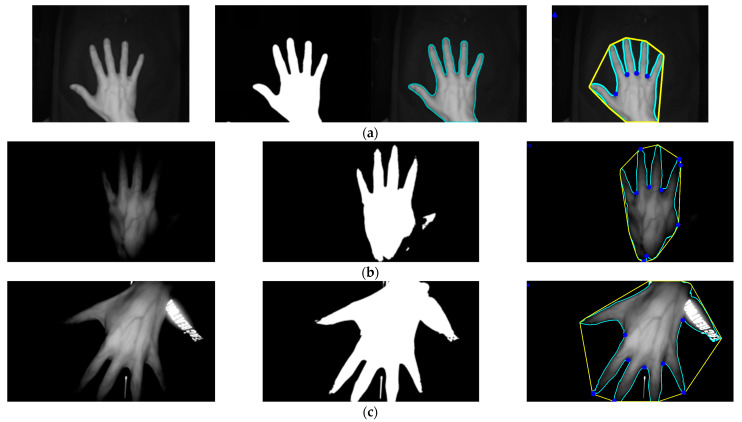
The convex hull and convex method to detect keypoints in the Jilin University public dataset and self-built dataset. (**a**) Detection effect of the Jilin University dataset. Self-built dataset: (**b**) detection effect under incomplete occlusion; (**c**) detection effect when the background contained reflections of clothing.

**Figure 16:**
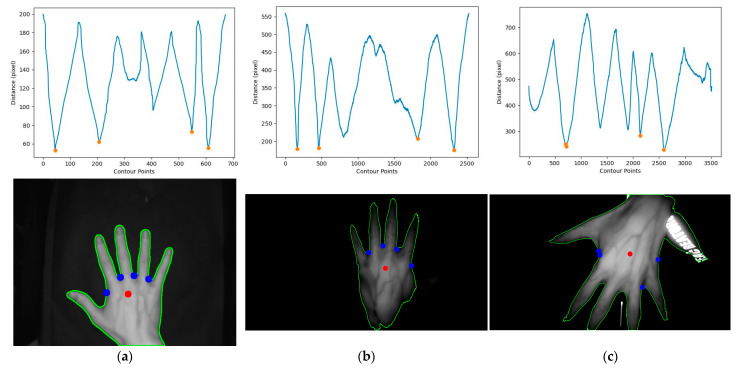
The centroid distance method to detect keypoints in the Jilin University public dataset and self-built dataset. (**a**) Detection effect of the Jilin University dataset. Self-built dataset: (**b**) detection effect under incomplete occlusion; (**c**) detection effect when the background contained reflections of clothing.

The first method involved converting the original image to a grayscale image followed by binarization. The binarized image was then analyzed for its contours, and the convex hull and convex defects of the contour around the hand were detected to locate the keypoints. The second method also began with converting the original image to grayscale and binarizing it. The hand’s contour was analyzed to calculate its centroid, and all points on the contour were measured for their distance from the reference point. The four closest points were then selected as the keypoints. Both of these traditional image processing methods were used to locate keypoints in the Jilin University dataset. The implementation and effectiveness of the convex hull and convex defect detection method can be seen in Figure 15, while the effectiveness of the centroid distance method can be seen in Figure 16. After using the same keypoint detection method for the self-built dataset and Jilin University dataset, it can be seen in Figure 15 and Figure 16 that the traditional image processing method could achieve good keypoint detection with a clean background and the hand intact but with incomplete occlusion due to shadows caused by the bending of the dorsal hand and interference in the background, so binarizing the image could not generate the correct binary image of the dorsal hand. This resulted in the inability to detect the correct keypoints after contour detection and detection of convex hulls and convexity defects. The centroid distance method detected more than four local minima in complex backgrounds. Two traditional image processing methods as well as the keypoint detection method proposed in this study are presented in Table 5 with their respective accuracies. Complex backgrounds caused the traditional image processing methods to fail completely.

### 3.8. Hand Dorsal Vein Verification Experiment

**Figure 17:**
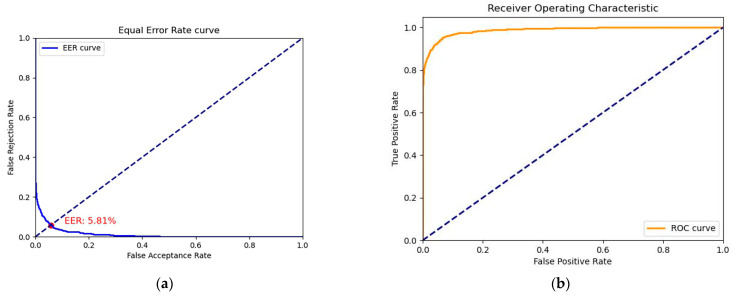
The equal error rate (EER) and receiver operating characteristic (ROC) curve for the task of dorsal hand vein authentication: (**a**) EER curve; (**b**) ROC curve.

The improved U-Net model was used to extract regions of interest (ROIs) from hand dorsal vein images in a self-built dataset of 359 classes, where each hand was considered as a separate class. The dataset was split into training and testing sets at a ratio of 7:3. The MobileNetV2 model was chosen for classification training. After training, the fully connected layer used for classification was removed from the MobileNetV2 model, and the model was used to extract feature vectors for the ROIs. Each image in the test set was subjected to one-to-one verification, and the equal error rate (EER) and ROC curve were calculated. The results are shown in Figure 17.

**Figure 18:**
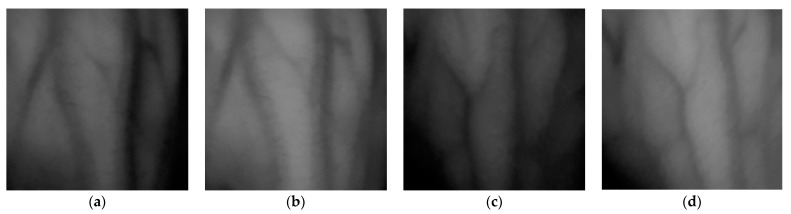
Partial sample with uneven illumination: (**a**,**b**) are hand vein images of the same user, while (**c**,**d**) are hand vein images of another user.

Figure 18a,b depict the dorsal hand vein pattern of the same user, while Figure 18c,d depict the dorsal hand vein pattern of another user. Both the training and testing sets were unprocessed, and due to the non-restrictive imaging conditions (i.e., the hand posture was not strictly required to be parallel to the imaging device, and the hand position was not strictly required to be centered in the image), the uneven illumination caused by different hand positions led to a high EER of 5.81% in the verification results. This study mainly focused on improving the U-Net model for detecting key points to extract the ROI. Therefore, we only reported simple results for dorsal hand vein verification experiments. In future work, we will conduct in-depth research on the performance of feature vectors extracted from neural networks and traditional feature descriptors for dorsal hand vein ROI extraction in order to solve the problem of low robustness caused by uneven illumination under unconstrained conditions.

## 4. Conclusions

This study proposed a method for dorsal hand keypoint detection based on deep learning and extracting regions of interest to target the difficulty of traditional image processing algorithms in locating key points in non-contact dorsal hand vein images. Based on the U-Net network structure, residual modules were added to the downsampling path of the U-Net network, and the transpose convolutional layers in the upsampling path of the U-Net network were improved to avoid the checkerboard effect by using bilinear interpolation. The JS divergence loss function was used to supervise the final feature map distribution, and Soft-argmax was used to calculate the keypoint coordinates from the final feature map for end-to-end training, which facilitated network model training.

(1) The proposed improved U-Net network achieved an accuracy of 98.6%, which was 1% higher than that of the original U-Net network model. The file size of the improved U-Net network model was 1.16 M, which was nearly 1/30 that of the original U-Net network model. Therefore, the proposed method achieved a higher accuracy than the original U-Net network model while significantly reducing the number of model parameters, making it suitable for deployment in embedded systems.
(2) The experiments on the self-built dataset and the Jilin University dataset showed that the model could achieve a high accuracy in both complex and clean backgrounds and can meet the requirements of practical applications in real environments.

## Figures and Tables

**Table 1:** Model comparison tests for improved U-Net models with and without short-circuit connections.

Network Model	Reasoning Time/ms	Accuracy Rate/%	Model Size
No short-circuit connection	42.48	98.0	1.14 M
With short-circuit connection	47.57	98.6	1.16 M

**Table 2:** Model comparison tests for improved U-Net models using transposed convolution and bilinear interpolation.

Network Model	Reasoning Time/ms	Accuracy Rate/%	Model Size
Transposed convolution	56.32	96.7	1.18 M
Bilinear interpolation	47.57	98.6	1.16 M

**Table 3:** Comparison trials of improved U-Net models trained with different losses.

Mean Squared Error (MSE) Loss	Jensen–Shannon Divergence Loss	Euclidean Distance Loss	Accuracy Rate/%
√			92.3
	√		95.0
		√	95.5
√	√	√	98.6

**Table 4:** Comparison tests of the U-Net model before and after improvements.

Network Model	Reasoning Time/ms	Accuracy Rate/%	Model Size	Total Floating Point Operations per Second (FLOPs)
U-Net	146.12	97.6	35.0 M	7.44 G
Improved U-Net	59.57	98.6	1.16 M	939.0 M

**Table 5:** Detection accuracy of traditional image processing methods and improved U-Net.

Detection Method	Self-Built Dataset	Jilin University Dataset
	Accuracy Rate/%	Accuracy Rate/%
Convex hull and convex	55.0	99.0
Centroid distance	69.1	97.9
Improved U-Net	98.6	99.5

## Data Availability

Not applicable.
